# Recurrence of COVID-19 infection symptoms in short time; reinfection or reactivation? Three cases of three healthcare workers and a literature review

**DOI:** 10.1016/j.amsu.2022.104619

**Published:** 2022-09-13

**Authors:** Nadeem Ahmed, Kinan Abbas, Ebrahim Makhoul, Rudy Deeb, Ashraf Raya, Nagham Khaddour, Faisal Redwan

**Affiliations:** a Department of Cardiovascular Diseases, Tishreen University Hospital, Latakia, Syria; b Department of Gastrointestinal Diseases, Tishreen University Hospital, Latakia, Syria; c Tishreen University Hospital, Latakia, Syria; d Department of Neurology, Tishreen University Hospital, Latakia, Syria; e Department of Internal Medicine, Tishreen University Hospital, Latakia, Syria; f Head of Laboratory Medicine Department and Assistant Professor of Clinical Biochemistry, Tishreen University, Latakia, Syria

## Abstract

**Background:**

Since the beginning of the COVID-19 pandemic, many research papers have been published focusing on some recurrence cases of symptoms after a long period of free symptoms with a negative RT-PCR retest. There is no crucial evidence until now of the possibility of recurrence, immune system reactivation, or reinfection.

**Methods:**

Three cases of resident doctors who recovered from COVID-19 but represented symptoms with new positive RT-PCR were discussed. Clinical data, laboratory tests, RT-PCR results, and antibodies titers all were collected. Moreover, many cases from the literature have been reviewed and compared.

**Results:**

The long-term exposure has not succeeded in forming an effective immune response, especially, since they do not have any significant history of chronic illnesses or a diagnosed immune disorder. While the antibody response occurred only in the second patient, it did not prevent new infection, but did it control the severity of the infection or its complications?

**Conclusion:**

Our three patients are health workers and have been in direct contact with COVID-19 patients. The inflammatory response parameters may not be reliable in predicting the activation of the immune response and the formation of the antibodies. We still need to find answers for reactivation and reinfection issues.

## Highlights

• A direct contact with COVID-19 patients failed in forming an effective response.
• The antibodies controlled the severity, complications and inflammatory response.
• The correlation between the severity of symptoms and laboratory markers.

## 1 Introduction

COVID-19 started in December 2019 in Wuhan, China, and reached quickly all parts of the world [1,2]. Still, there is an evidential lack in how the immune system responds. Many papers have reported that some COVID-19 patients had positive RT-PCR tests after they were discharged from hospitals with negative RT-PCR [1,3,4]. Since the beginning of the pandemic, many research papers have been published focusing on some recurrence cases of COVID-19 symptoms after a long period of free symptoms with negative RT-PCR tests. Clinical and laboratory differences between the two episodes of recurrence, the medical history of the patients and the range of their exposure especially if they are health workers were discussed to obtain a clear description of these cases whether they are reactivation or reinfection cases. There is no crucial evidence until now of the possibility of recurrence, immune system reactivation, or reinfection [3,4]. Through our paper, we are presenting three cases of resident doctors who recovered from COVID-19 but represented symptoms with new positive RT-PCR. The purpose of this paper is to ask the following questions: Why has not the long-term exposure succeeded in forming an effective immune response? Can Inflammatory Response Parameters be reliable in predicting the activation of the immune response and the formation of the antibody response? We also encourage doing more research about the correlation between the severity of symptoms and laboratory markers (see Table 1, Table 2, Table 3).

**Table 1:** Laboratory tests for the first patient.

	30/11/2020	11/01/2021
WBC 10^3^/mm^3^	6500	4800
NEUT 10^3^/mm^3^	3800	3300
LYM 10^3^/mm^3^	2500	***1200***
HGB g/dL	13.8	15.1
PLT 10^3^/mm^3^	218	184
CRP U/L	1.8	5.81
AST U/L		14
ALT U/L	11	10.4
CREAT mg/dL	0.7	0.87
CK U/L		54.2
DDIMER ng/ml	79.59

**Table 2:** Laboratory tests for the second patient.

	26/09/2020	28/12/2020
WBC 10^3^/mm^3^	4500	5700
NEUT 10^3^/mm^3^ rowhead	2600	4500
LYM 10^3^/mm^3^	***1500***	***800***
HGB g/dL	15.5	15.9
PLT 10^3^/mm^3^	***128***	***146***
CRP U/L	***7.21***	1.83
AST U/L	26.5	
ALT U/L	26.8	20.3
CREAT mg/dL	1.07	1
UREA mg/dL	23	22
CK U/L	140.2	
DDIMER ng/ml	123

**Table 3:** Laboratory tests for the third patient.

	12/08/2020	11/12/2020
WBC 10^3^/mm^3^	6300	8100
NEUT 10^3^/mm^3^	3000	5600
LYM 10^3^/mm^3^	2700	1700
HGB g/dL	13.3	13.2
PLT 10^3^/mm^3^	179	216
CRP U/L	2.08	6.1
AST U/L	***42.7***	
ALT U/L	***74.1***	14
CREAT mg/dL	0.74	0.7
UREA mg/dL	20	15
CK U/L	56.8	

In addition, we are searching for practical ways to detect the infectious cases and the reliability of symptoms severity, symptoms timing, and the inflammatory response parameters. This will help us determine the recurrence state and predict the activation of the immune response and the formation of the antibody response in developing countries where are no accurate genome studies.

## 2 Methods

We are discussing three cases of resident doctors at our hospital who recovered from COVID-19 but represented symptoms with new positive RT-PCR. Clinical data, laboratory tests, RT-PCR results, and antibodies titers all were collected using ELISA techniques for antibodies titers, LightCycler from Roche for RT-PCR testing, and Mindray machine for blood chemistry analysis.

Moreover, we have searched the net using Pubmed and Google scholar search engines to find similar cases of healthcare providers who got reactivated or reinfected by COVID-19. We discussed similar cases and included some other studies discussing the same issue.

## 3 Results

**Fig. 1:**
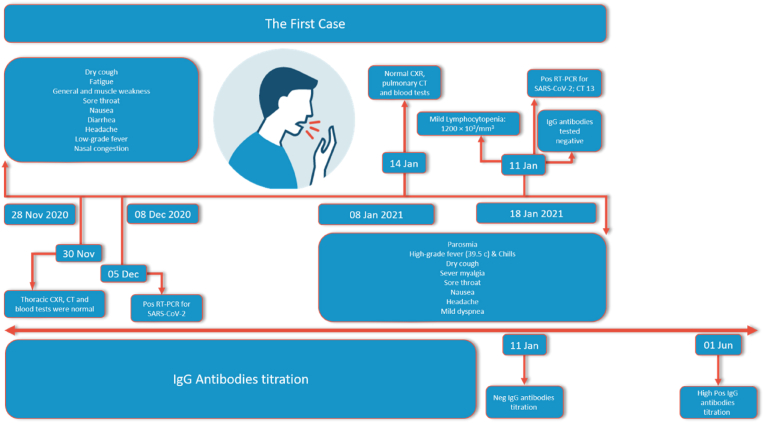
Clinical scenario for the first patient.

Case 1: A 25-year-old male resident doctor with a clean medical history presented symptoms on November 28, 2020. On December 5, 2020, the RT-PCR of a nasopharyngeal swab tested positive for SARS-CoV-2. Supportive treatment was taken without the need for hospitalization. On January 8, 2021, the second episode of symptoms was presented. Most of the symptoms lasted for 10 days. RT-PCR was positive for SARS-CoV-2 at the same laboratory with a CT value of 13. The time between the onsets of symptoms in both episodes was about 42 days. Covid-19 IgG antibodies tested negative twice: on January 11 and February 28. However, another titer was done on June 01, 2021, with a high-positivity result. His clinical scenario is presented in Fig. 1.

**Fig. 2:**
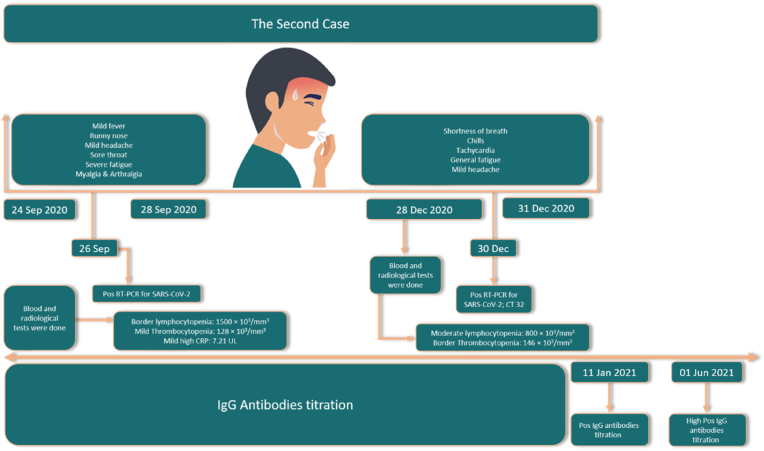
A clinical scenario for the second patient.

Case 2: A 26-year-old male resident doctor with a medical history of only a tonsillectomy. The first scenario was on September 24, 2020, after an extensive exposure during ER shifts. All the symptoms lasted for 4 days. A nasopharyngeal swab was taken on September 26, 2020, and a positive RT-PCR confirmed the infection. A new symptoms onset was on December 28, 2020, with an interval of 94 days between the two events. A nasopharyngeal swab was taken and RT-PCR tested at the same laboratory and came out positive with a CT value of 32. Total clinical recovery was documented three days later. There was no need for hospitalization with the two events. Covid-19 antibody test was conducted on January 11, and showed a slight-positivity for IgG-antibodies, with another high positive result was tested on June 01, 2021. The clinical scenario is presented in Fig. 2.

**Fig. 3:**
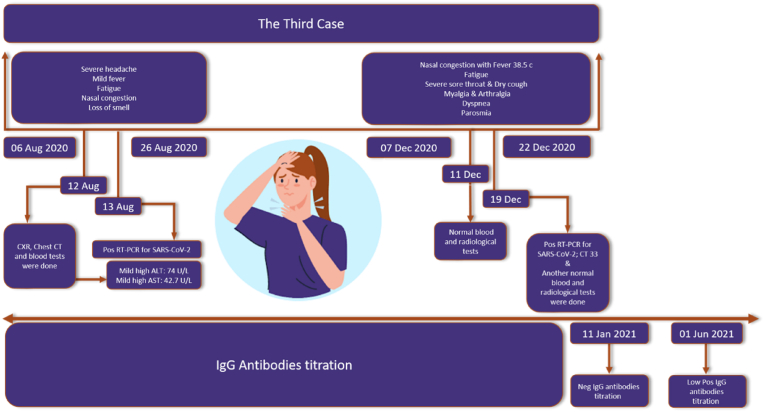
Clinical scenario for the third patient.

Case 3: A 26-year-old female doctor presented symptoms on August 6, 2020, and lasted 15 days. On August 13, a nasopharyngeal swab was taken and RT-PCR was positive. 4 months later, on December 7, 2020, new clinical symptoms were presented again and lasted for 15 days. On December 19, a nasopharyngeal swab was taken and RT-PCR was tested at the same laboratory and came out positive with a CT value of 33. COVID-19 antibody test was conducted and showed negativity for IgG-antibodies. The other two titers were tested later and showed slight positive results. The clinical scenario is presented in Fig. 3.

## 4 Discussion

The difference between prolonged shedding which leads to reactivation and real reinfection should be distinguished. One of SARS-CoV-2 features is prolonged virus shedding [5]. One of the first reported cases of recurrence of clinical manifestations was for 6 health workers from Brazil, between March 16, 2020, and April 19, 2020, when symptoms reappeared with a positive RT-PCR during 53–70 days between the two events. Three of them had a negative RT-PCR between the two events. Two of them needed admission to the Intensive Care Unit for the second time [6]. At the time, it was not possible to ascertain whether these cases were reinfections or reactivations. Reactivation is a re-detectable positive SARS-CoV-2 viral RNA in a recovered patient that occurs within the first 4 weeks of the previous infection, while reinfection occurs when the time lag between discharge and the re-detectable positive SARS-CoV-2 viral RNA is at least 28 days [7]. For SARS-CoV-2, reinfection appears to be uncommon during the initial 90 days after symptoms onset [8]. Centers for Disease Control and Prevention (CDC) distinguishes between two main scenarios of cases during the 90 days: the first is the development of new positive SARS-CoV-2 RT-PCR without new symptoms after the first incidence, which is more likely to represent persistent shedding of viral RNA than reinfection. The second is the development of new symptoms during the 90 days without any other identified diagnosis other than Covid-19 infection, which highly recommends the reinfection of SARS-CoV2.

Different methods were used to detect reinfection cases. Genome sequencing is the best to identify the reinfection [9,10], but it is limited by many challenges, especially, the lack of banking samples of primary infection [10]. Other concepts could be used to make the reinfection more considerable than the reactivation, such as the time lag between the two presentations, symptoms severity, and RT-PCR tests with Ct value. The CDC sets a cut-off CT value of 33 with positive RT-PCR 90 days after the first infection or 45 days after the first infection with compatible symptoms or epidemiological exposure [10]. Small centers cannot use genome sequencing due to its high expenses and the need for expertise. Therefore, they are restricted to the ways mentioned above. There is not enough information about the time needed to form the antibodies and immune response mechanism in the primary COVID-19 infection, recurrence, or reactivation. Does the severity of clinical symptoms relate to antibodies formation? Do laboratory changes relate to antibodies formation? Does the presence of antibodies cause a less severe infection? Studies in animal models have shown the presence of the modified antibodies assesses sepsis and disease and impairs the susceptibility of the virus to replication in the respiratory epithelium [11,12]. In the 16 reinfection cases confirmed by genome sequencing, with an average of 66 days (19–142 days) between the first and second infection, positive antibody titers were documented in 10 of them with the onset of the second infection [10]. An interval of 142 days between two infections in one published clinical case from Hong Kong was analyzed by genome sequencing. The first was symptomatic and the positive antibody response was not recorded until the fifth day after the second infection, 145 days after the first one. A negative antibody titration was presented 10 days after the first infection [13]. Did the antibody response start later because of the first infection? Was the first infection the reason behind the asymptomatic second infection? Or did it occur as a result of an immune enhancement?! In contrast, in another case in Nevada in the USA, reinfection was confirmed by genome sequencing occurred after 64 days, and it was severe with hypoxia and significant respiratory failure with a need for hospitalization. The antibody response was recorded 8 days after the beginning of the reinfection, 73 days after the first infection [14]. Was this due to a more virulent virus strain [15], a very high viral load, or antibodies response enhancement [16]? A case from Ecuador showed a second infection to be more severe than the first, but cases from Belgium, the Netherlands [17], and Hong Kong [18] did not show a difference between the severities of the two events. Reviewing the published cases confirmed as reinfection by genome sequencing. 9 out of 12 cases were asymptomatic or had mild primary sepsis. The rest were moderate to severe. In the 12 cases, the first infection was compared with the second. Half of the cases had less severe clinical scenarios, which promotes the idea of the effectiveness of the first infection in activating partial protection, and contrasts the idea of the immune enhancement dependent on the antibody response seen in other viral pathogens. In cases with the primary infection moderate to severe, high antibody titers were noted, which suggests subsequent protection influenced by the first severe infection. Determining the CT value is useful in giving information about the severity of the infection, and comparing its value between the two events helps in predicting if the cases are reinfections or reactivations. 14 of these cases were recorded with a CT value in the two events. The value average in the first was 32.5 (17–38) and in the second was 27.3 (16–39.6). Maybe the case is more complicated for health workers due to the exposure to a high viral load. Two health workers from India developed two confirmed infections with a positive RT-PCR. The difference between the two infections is 107 days for the first and 109 days for the second. Both infections are asymptomatic. For the first patient, the CT values were 36 and 16.6 for the first and second infection, respectively; and for the second one, 28.16 and 16.92 for the first and second infection, respectively [19].

In our cases, both the second and third patients developed new clinical symptoms after more than 90 days of the first infection, with a positive RT-PCR and CT values 32 and 33, respectively. We found that the first patient developed new clinical symptoms within only 40–45 days from the first infection with a positive RT-PCR test, a CT value of 13, and intensification of symptoms in general in the second infection that lasted for about 10 days. For the first patient and according to the CDC guidelines, no negative RT-PCR between the two infections, the period of clinical remission between the two infections, and the severity of symptoms suggest reinfection rather than reactivation. That is supported by the negative titration of specific antibodies after 75 days from the first infection, and the second titration during 48 days after the second one. Both the first and third patients reported an exacerbation of symptoms the second time with a longer duration of the symptomatic period. Both presented negative titration of specific IgG antibodies within 75 days after the first clinical infection of the first patient, and 160 days after the first clinical infection of the third patient. However, the second patient did not report any exacerbation of symptoms the second time, and he recorded an antibody response within 109 days from the first infection and 14 days from the second one.

We have observed that the three cases were similar with mild symptoms the first time, and significant clinical exacerbation the second time (except for the second patient who recorded a positive antibody response). In addition, only the second patient recorded laboratory changes represented in the first infection with a slight decrease in the lymphocyte count of 1500 with a decrease in platelets 128,000 and a slight rise in CRP 7.2 with an obvious lymphopenia of 800 in the second infection. The follow-up showed important complications for both the first and third patients on exercise tolerance. The first patient had repeated isolated sore throats without any additional symptoms, and it recurred at a rate of 2–3 times within 3 months, and each did not last more than 3–4 days. At that time, RT-PCR smear was performed with a negative result. Moreover, a high positive antibody titer was recorded in the end.

The second patient was completely vaccinated with two doses of Covid-19 vaccine and then developed a significant antibody response for about 60 days in addition to the previous initial response. The third patient, who has not received the vaccination, mentioned mild clinical symptoms after about 5 months from the second confirmed infection accompanied by a negative RT-PCR. By investigating the antibody response again, a mild result was noted, but with retesting after about 30 days, a slight regression in the antibody response was recorded.

This study has two important limitations that should be noted. First, there was no negative RT-PCR tests during the period between the two symptomatic scenarios of each case, and we totally relyed on symptoms progression. Second, we do not have centers to perform the genome sequencing as a developing country with low economical sources, but we are searching for practical criterion to detect the development of cases.

## 5 Conclusion

Through our paper, we are asking important questions: our three patients are health workers and have been in direct contact with COVID-19 patients. Why has not the long-term exposure succeeded in forming an effective immune response, especially, since they do not have any significant history of chronic illnesses or a diagnosed immune disorder? Can Inflammatory Response Parameters be reliable in predicting the activation of the immune response and the formation of the antibody response? We are assuming the antibody response that occurred in the second patient did not prevent a new infection, but it controlled the severity of the infection, its complications, and the inflammatory response.

We encourage doing more research to find the best management methods for COVID-19 pandemic as it showed the importance of having continues evaluation and concrete management [21,22]. We should find the correlation between the severity of symptoms and laboratory markers. More research is needed to know if the clinical and laboratory findings are enough to determine the immune response to COVID-19 and the anti-bodies formation, especially, in the developing countries due to the limitation of using expensive examinations like genome sequencing. Moreover, the COVID-19 pandemic had negative socio-economic implications that should be considered [20]. We also need to know how important to the health workers who have been infected to make tests to determine the antibodies formation and the probability of the reinfection occurrence and its intensity.

## Provenance and peer review

Not commissioned.

## Ethical approval

Informed consent was obtained from the patient regarding the report of their clinical scenario data in an anonymous way.

## Sources of funding

No source of funding.

## Author contribution

Nadeem Ahmed: drafted, discussed, edited and supervised the authors. Kinan Abbas: reviewed and compared the three cases with the literature. Ebrahim Makhoul: drafted and searched for similar cases in the literature. Rudy Deeb, Ashraf Raya and Nagham Khaddour: Participated in collecting the data. Faisal Redwan: the guarantor and supervisor, critically revised the article and approved the final manuscript.

## Conflicts of interest

No conflicts of interest.

## Registration of research studies

1. Name of the registry: Recurrence of COVID-19 infection symptoms; reinfection or reactivation? A case series of three health workers and a literature review.
2. Unique Identifying number or registration ID: researchregistry8103
3. Hyperlink to your specific registration (must be publicly accessible and will be checked): https://www.researchregistry.com/register-now#user-researchregistry/registerresearchdetails/62d17d93b02a270020ee609f/

## Guarantor

Faisal Redwan: Head of Laboratory Medicine Department and lecturer of Clinical Biochemistry, Tishreen University, Latakia, Syria.

## Consent

Written informed consent was obtained from the patient for publication of this case report and accompanying images. A copy of the written consent is available for review by the Editor-in-Chief of this journal on request.
